# Effects of Diazepam on Reaction Times to Stop and Go

**DOI:** 10.3389/fnhum.2020.567177

**Published:** 2020-10-06

**Authors:** Swagata Sarkar, Supriyo Choudhury, Nazrul Islam, Mohammad Shah Jahirul Hoque Chowdhury, Md Tauhidul Islam Chowdhury, Mark R. Baker, Stuart N. Baker, Hrishikesh Kumar

**Affiliations:** ^1^Department of Neurology, Institute of Neurosciences Kolkata, Kolkata, India; ^2^Department of Physiology, University of Calcutta, Kolkata, India; ^3^Department of Neurology, National Institute of Neurosciences and Hospital, Dhaka, Bangladesh; ^4^Department of Neurology, Royal Victoria Infirmary, Newcastle upon Tyne, United Kingdom; ^5^Department of Clinical Neurophysiology, Royal Victoria Infirmary, Newcastle upon Tyne, United Kingdom; ^6^The Medical School, Newcastle University, Newcastle upon Tyne, United Kingdom

**Keywords:** benzodiazepine, Diazepam, SSRT, motor stopping, GABA

## Abstract

**Introduction**: The ability to stop the execution of a movement in response to an external cue requires intact executive function. The effect of psychotropic drugs on movement inhibition is largely unknown. Movement stopping can be estimated by the Stop Signal Reaction Time (SSRT). In a recent publication, we validated an improved measure of SSRT (optimum combination SSRT, ocSSRT). Here we explored how diazepam, which enhances transmission at GABA_A_ receptors, affects ocSSRT.

**Methods**: Nine healthy individuals were randomized to receive placebo, 5 mg or 10 mg doses of diazepam. Each participant received both the dosage of drug and placebo orally on separate days with adequate washout. The ocSSRT and simple reaction time (RT) were estimated through a stop-signal task delivered *via* a battery-operated box incorporating green (Go) and red (Stop) light-emitting diodes. The task was performed just before and 1 h after dosing.

**Result**: The mean change in ocSSRT after 10 mg diazepam was significantly higher (+27 ms) than for placebo (−1 ms; *p* = 0.012). By contrast, the mean change in simple response time remained comparable in all three dosing groups (*p* = 0.419).

**Conclusion**: Our results confirm that a single therapeutic adult dose of diazepam can alter motor inhibition in drug naïve healthy individuals. The selective effect of diazepam on ocSSRT but not simple RT suggests that GABAergic neurons may play a critical role in movement-stopping.

## Introduction

Real-life environments require us to build or adapt different movement control strategies to accomplish a task goal or to respond rapidly to a fast-moving visual and/or auditory stimulus. During our engagement in these complex scenarios, we must be able to prioritize different actions (Mückschel et al., 2014). Response inhibition (or movement stopping) is a key component of executive control, providing the ability to suppress an action that has already been initiated but which is no longer required (Logan et al., 1984). Day-to-day life has numerous examples where such response control is needed, for example avoiding touching a hot pan, or stopping before crossing a road when a car is approaching at speed. A range of psychopathological and impulse control disorders severely impair response inhibition, for example, attention-deficit/hyperactivity disorder, obsessive-compulsive disorder, substance abuse, pathological gambling, and eating disorders (Bechara et al., 2006). Experimental studies in patients have helped to define the contribution of subcortical structures to response inhibition, specifically fronto-basal interactions (Whelan et al., 2012). Evidence from these studies suggest that the connection between supplementary motor area/inferior frontal gyrus and sub-thalamic nucleus (Inase et al., 1999; Aron et al., 2007) is crucial in controlling response inhibition (Aron and Poldrack, 2006; Frank, 2006; Li et al., 2008; Hikosaka and Isoda, 2010; Munakata et al., 2011; Forstmann et al., 2012).

The stop-signal paradigm is well-suited for laboratory investigation of response inhibition. Participants perform a reaction time (RT) task in response to a Go cue. Occasionally, the Go signal is followed by a stop signal after a variable delay (the stop signal delay). Using the probability of an inappropriate response after the stop signal, and the distribution of RTs on Go trials, this paradigm allows estimation of the covert latency of the stopping process, or stop signal reaction time (SSRT). This has been used extensively to explore the cognitive and neural mechanisms of response inhibition (Hanes and Schall, 1996; Aron and Poldrack, 2006; Verbruggen et al., 2014; Debey et al., 2015). Studies with SSRT have found correlations between individual differences in stopping and behavior such as risk-taking, substance abuse, and control of impulses/urges (Schachar and Logan, 1990; Ersche et al., 2012; Whelan et al., 2012). Moreover, movement stopping can be enhanced or impaired by a variety of factors. The drug methylphenidate enhances stopping (Tannock et al., 1995), whereas by contrast, in long-term users, cocaine impairs response inhibition (Fillmore et al., 2002). Increased motivational incentives can enhance stopping (Boehler et al., 2014).

Recently we have developed an improved index by applying Bayesian statistics to SSRT estimation. This index, which appears to have significantly higher reproducibility (Choudhury et al., 2019), is known as optimum combination SSRT (ocSSRT).

Those who abuse drugs often develop impairments in performance and attention, and increases in impulsive behavior (Heishman et al., 1997; De Wit and Richards, 2004). Evidence from animal studies has shown that D2 receptors are essential both for psychostimulant activity and motor response inhibition (Dalley et al., 2007). Methylphenidate is a dopamine and noradrenaline reuptake inhibitor; it has varied effects on movement stopping, not all of which are reversed by blocking dopaminergic receptors (Eagle et al., 2007). This suggests that other monoaminergic transmitters may also play a role in motor response inhibition. In the cerebral cortex, around 10–15% of neurons in the cerebral cortex are GABAergic inhibitory interneurons, which can be sub-divided into multiple cell types (Ascoli et al., 2008). In the STN, around 7.5% of cells are GABAergic (Lévesque and Parent, 2005). The major components of the known neural circuitry for response inhibition (Aron et al., 2014) should therefore be susceptible to GABAergic modulation. Diazepam is a widely prescribed anxiolytic, muscle relaxant, and anticonvulsant that is commonly abused (Woods et al., 1987; Gelkopf et al., 1999). Diazepam administered at standard therapeutic doses (5–10 mg) reportedly did not affect measures of behavioral inhibition including delay discounting, a Go/No-Go task, or the stop signal reaction task, despite the drug-producing prototypical sedative-like effects (Reynolds et al., 2004). At higher doses (20 mg) it did impair performance on both Go/No-Go and stop-signal tasks but did not affect measures of delay discounting (Acheson et al., 2006). Here, we aimed to assess the impact of a benzodiazepine on movement stops. We conducted a randomized, placebo-control, cross-sectional, double-blinded trial, which compared the effect of two therapeutic doses of diazepam (5 and 10 mg) on the improved novel measure ocSSRT.

## Materials and Methods

### Population and Trial Protocol

**Figure 1:**
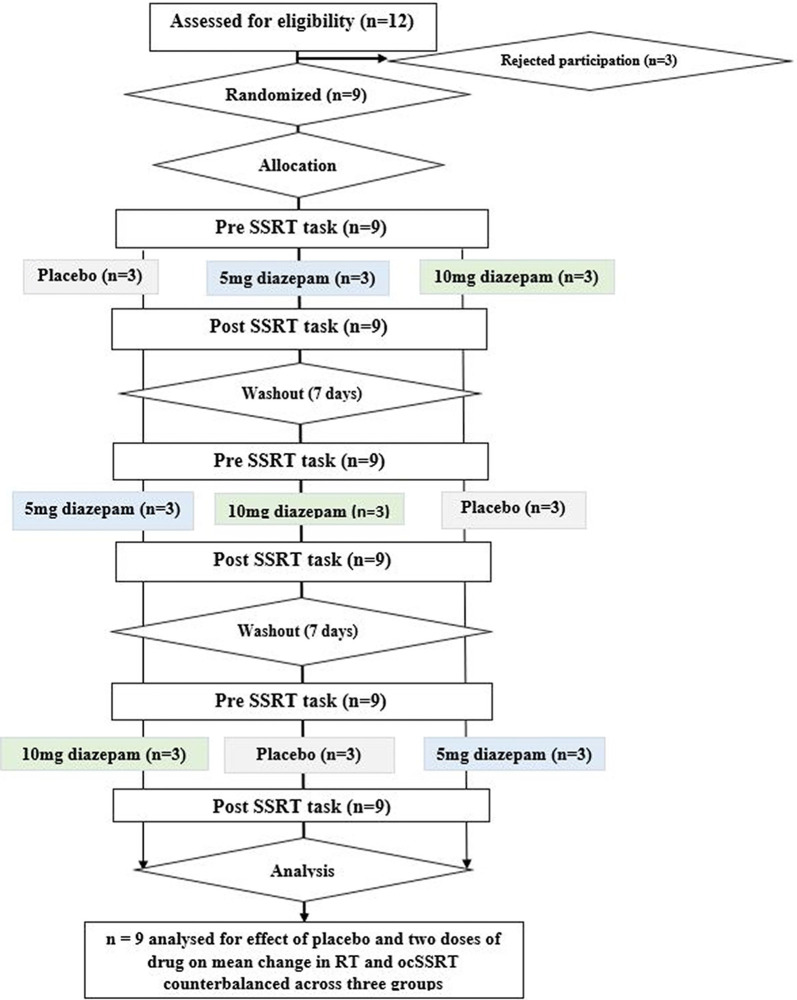
Consort diagram for the study, depicting randomization, group allocation, washout periods, and data analysis.

Twelve potential participants were initially screened for this pilot study. We did not perform a prior power calculation to determine the number of participants, as we had no information on the expected effect size. Three were excluded as one had a drug allergy, one was taking a benzodiazepine as a medication already, and one discontinued because of personal reasons. The nine remaining subjects came from our research laboratory (five male and four female, including two authors of this report; age 27 ± 4 years, mean ± SD), all educated to post-graduate degree level or higher with no known underlying neurological disorder, and were randomly assigned to three groups. The order in which the three conditions (placebo; 5 mg diazepam; or 10 mg diazepam) were recorded was counterbalanced across groups. Study participants received no financial compensation. Each participant received both the dosage of drug and placebo orally on separate days with an adequate washout interval (1 week). The estimation of ocSSRT was performed through a stop-signal task (see below), immediately before and 1 h after dosing (peak plasma concentration of diazepam is achieved approximately 1 h after ingestion, Mandelli et al., 1978). The trial protocol is summarized schematically in Figure 1. We measured ocSSRT and RT, where ocSSRT represents movement stopping and RT is the simple response time.

Experiments were conducted in a tertiary care neurology center in Eastern India. Written informed consent was obtained from each participant before the study following the Declaration of Helsinki. Protocols and procedures were approved by the Institutional Ethics Committee (reference number I-NK/IEC/99/2019 ver.1. dated 29 April 2019) and the trial was registered prospectively with the Clinical Trials Registry-India (CTRI), registration number CTRI/2020/02/023530 on 24 February 2020.

### Device

We used a custom-built battery-powered device housed in a plastic case, which the subject held comfortably in two hands (Choudhury et al., 2019). One red and one green light-emitting diode (LED, 5 mm diameter) mounted on the front of this box indicated Stop and Go respectively; a press button (2 cm diameter) positioned beneath the LEDs was depressed and held or released by the subject depending on the instructions encoded by the sequence of LED flashes. A four-line liquid crystal display (LCD) screen providing a textual status display during the test was positioned above the LEDs. A microcontroller (dsPIC30F6012A, Microchip Inc.) programmed with custom firmware written in C using the MPLAB development environment within the device determined the task sequence, measured RTs (1 ms precision) and response probabilities, and computed the SSRT. The Task outcome as a numerical value of the estimated ocSSRT and RT was then displayed on the LCD screen and copied to a laboratory notebook, and thence to a spreadsheet, by the experimenter. The device did not keep a permanent record of single-trial responses.

Mathematical details of the calculation of ocSSRT are provided in Choudhury et al. (2019), which should be consulted for a full description. Briefly, for a given stop-signal delay (SSD), the number of inappropriate responses *M* and the total number of trials tested with that delay *N* was determined. Instead of simply estimating response probability *p* as *M*/*N*, a Bayesian approach was used to estimate the likelihood of a particular response probability *p*, assuming that the response number M followed a binomial distribution. To calculate SSRT for a particular response probability *p*, we found the point in the distribution of RTs to a Go cue alone where a fraction *p* of RTs were smaller (*RT*) and subtracted the stop signal delay SSD, so *SSRT* = *RT* − *SSD*. This allowed calculation of the likelihood of a range of SSRT values. SSRT likelihood curves were found for each of the four SSD values, and then a combined SSRT likelihood curve computed from the product of the individual curves. The mean of this distribution gave the ocSSRT. This approach is an improvement over simpler approaches that average single estimates of SSRT for each SSD, as it naturally takes account of the reliability of each estimate.

### Detailed Test Procedure

Study participants were randomized to each interventional group by a computer-generated random sequence generator (Random Allocation, Ver. 2.0 software). Investigators and participants were blinded to the group allocation during the entire study period. The study drugs were dispensed by an unblinded study coordinator who was not involved in any of the assessment procedures or analyses. The placebo (ascorbic acid 500 mg) and the active compound (Valium 5 diazepam tablets, Abbott) had the same external appearance. To mask any differences in taste, participants were requested to swallow the tablets with a strongly flavored lemon drink.

All participants sat comfortably in a semi-illuminated, quiet room holding the task device. Participants were asked to respond to a Go cue as fast as they could, but to inhibit their responses on the trials when a Stop cue appeared. A trial was initiated by pressing and holding the response button with the index finger or thumb of the dominant hand (dominant side as subjectively reported by the participants). The LCD screen then showed the instruction “release on the green, hold on red.” The green LED illuminated after a delay (chosen from a uniform random distribution between 1 and 2.638 s). No other LED illuminated on 75% of trials, and the subject was required to release the button to respond (a Go trial). In 25% of trials, the green LED extinguished and the red LED illuminated (a Stop trial). For correct performance, the subject was required not to release the button. Four different SSDs (between the illumination of green and red LED) were used: 5 ms; 65 ms; 130 ms; and 195 ms. Trials were presented in blocks of 32, with 24 Go trials and eight Stop trials (two for each delay) within a block. The order was adjusted so that a Stop trial was always preceded and followed by a Go trial. There was a 1.3 s delay after each button release and before the next trial started. A Stop trial was considered successful if the button was not released for 0.7 s after the green LED illuminated; the next trial started after a 2 s delay. The task was paused for 60 s to allow the subject to rest after two blocks of 32 trials. Subjects could also pause the test at any point by releasing the button, as the next trial did not start until the button was depressed. Subjects sometimes did this for a few seconds, for example, to adjust their posture to be more comfortable, but did not choose to take longer rests other than at the scheduled times at the end of a set of 64 trials. One complete measurement typically lasted around 15 min. To aid with familiarization on the task, naïve subjects were allowed to complete 64 trials as practice; results from these were discarded.

The total duration of the study protocol was 2 weeks. During this time and 1 week before day one, the participants were not allowed to take any prescription or over the counter medications with potential neurotropic actions (e.g., anti-depressants, anxiolytics, sedatives, anti-tussives, common cold remedies).

### Statistical Analysis

Summary statistics of numerical variables were presented as mean and standard deviation (SD) for categorical variables. The normality of the data was tested using the Shapiro–Wilk test. Mean changes in RT, ocSSRT for placebo, 5 mg diazepam, and 10 mg diazepam were compared using repeated-measures ANOVA. Pairwise comparisons were completed by applying *post hoc* *t*-tests, with significance levels adjusted by a Bonferroni correction to account for the three comparisons (placebo vs. 5 mg diazepam, placebo vs. 10 mg diazepam, 5 mg vs. 10 mg diazepam). A corrected p-value of less than 0.05 was considered significant. All statistical analysis was performed using the SPSS 20 statistical package (SPSS, Chicago, IL, USA). As a pilot study, formal statistical calculation of sample size was not performed and convenience sampling was instead adopted.

## Results

**Figure 2:**
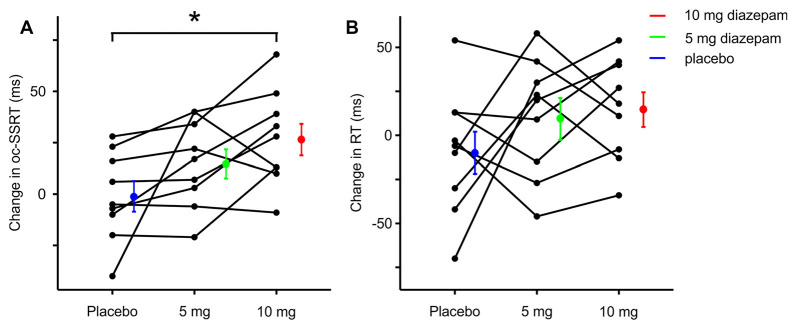
Change in ocSSRT and simple reaction time (RT) with placebo, 5 mg, and 10 mg diazepam. **(A)** Mean change in ocSSRT and **(B)** mean change in RT, in nine individuals. Significant differences from placebo are indicated by * (*p* < 0.05, *t*-tests).

Nine healthy individuals were randomly assigned to three groups, in which the order of testing the three conditions was counterbalanced. The baseline ocSSRT and RT (measured before ingestion of drug or placebo) were comparable between the three groups (Table 1). Subjects made inappropriate responses on between 0 and 73% of Stop trials, depending on the SSD. Changes in ocSSRT and RT from before to after placebo or drug ingestion were not distributed significantly differently from normal across the population (Shapiro–Wilk test statistic; *p* < 0.8 in all cases). Repeated measures ANOVA showed a significantly different effect of placebo and two doses of the drug on ocSSRT (Figure 2A; *F* = 6.790; *p* = 0.007). The further pairwise analysis revealed that the mean ocSSRT change (from baseline) was significantly higher with the 10 mg dose of diazepam compared to placebo (+27 ms vs. −1 ms, *p* = 0.012). ocSSRT also increased after 5 mg diazepam (mean change 15 ms), but this failed to reach statistical significance relative to placebo (*p* = 0.288).

**Table 1:** Comparison of baseline optimum combination Stop Signal Reaction Time (ocSSRT) and reaction time (RT) in three experimental sessions.

Baseline	Placebo	5 mg diazepam	10 mg diazepam	*p*-value
ocSSRT (ms)	190 ± 39	207 ± 44	204 ± 26	0.288
RT (ms)	408 ± 48	405 ± 27	412 ± 45	0.928

*Values are given as means ± standard deviation. *p*-values determined from repeated measure ANOVA*.

The change in RT from baseline (Figure 2B) was comparable after placebo, 5 mg and 10 mg of diazepam (mean changes −9, +10 and 15 ms, repeated measures ANOVA *F* = 1.399, *p* = 0.276; *t*-test between placebo and 5 mg, *p* = 0.867; *t*-test between placebo and 10 mg, *p* = 0.603).

It is important to consider the statistical power of our study, given the failure to detect a change in RT. We performed a *post hoc* power calculation for both ocSSRT and RT, using the measured standard deviation of the change in the experimental measure from before to after placebo (22 and 36 ms for ocSSRT and RT respectively), for a power level of 90%. With nine subjects, this indicated that we should detect a 27 ms change in ocSSRT, and a 45 ms change in RT, equivalent to 14% and 11% change respectively. We can therefore have confidence that any change in RT is likely to be smaller than this value.

Our results clearly show that even therapeutic doses of diazepam can affect stopping ability. Whilst we could not detect changes in a measure of simple motor response (RT), the ocSSRT was increased at the highest dose tested of 10 mg.

## Discussion

There are several ways to measure SSRT, and each may have advantages and disadvantages (Verbruggen et al., 2013; Leunissen et al., 2017). In this study, we used our recently-developed method exploiting portable equipment and an analytical approach which incorporates knowledge of the likely reliability of the response probability estimates. We have shown that this provides rapid SSRT measurements with high reproducibility (Choudhury et al., 2019). Regardless of any methodological differences, we can have high confidence in our results since this was a double-blind placebo-controlled trial, in which we demonstrated a significant difference between placebo and 10 mg diazepam.

Benzodiazepines are widely used psychotropic drugs. Medical indications for benzodiazepines are broad and include anxiety, insomnia, muscle relaxation, management of spasticity, and epilepsy. These drugs bind exclusively to and allosterically modulate GABA_A_ receptors (the major inhibitory receptor of the CNS), acting as partial agonists (Downing et al., 2005; Gielen et al., 2012; Möhler, 2015). Diazepam is a long-acting, medium potency benzodiazepine and is thus generally used for its anti-convulsive and anxiolytic effects. Long term use of diazepam has been associated with cognitive impairment, presumably as a side effect of the non-selective binding to all synaptic GABA_A_ subtypes (Rudolph and Knoflach, 2011). In the present study, we found that a single dose of diazepam impairs inhibitory control without significantly affecting RT.

Previously human studies showed that neural circuitry within the dorsolateral prefrontal cortex (Baker et al., 1996; Manes et al., 2002) and orbitofrontal cortex (Rogers et al., 1999) are involved in tests requiring planning and decision-making. Deakin et al. (2004) hypothesized that high doses of diazepam cause disinhibitory cognitive effects by impeding inhibitory networks within these cortical regions. They also speculated that diazepam can influence frontal lobe functions associated with decision making either by direct effects on GABA_A_ receptors within the frontal cortex or by modulating activity in the ascending reticular system (Deakin et al., 2004). In a rodent study involving the punished behavior model, Ford et al. (1979) showed that diazepam and d-amphetamine when administered in combination increased punished responding in all the rats. Ljungberg et al. (1987) evaluated the dose-dependent effects of diazepam on decision making in rats, in a rewarding behavior rodent model with water restriction paradigm. They observed that lever-pressing behavior in rats was not affected at a diazepam dose of 2 mg/kg but reduced significantly at doses of 5 mg/kg and 10 mg/kg. In a follow-up study, they found a selectively reduced tolerance of reward delay by diazepam (Ljungberg, 1990).

The phenomenon of response inhibition is not exclusively GABAergically mediated. Several rodent studies have shown that D2 receptor antagonism improves response inhibition when antagonists are infused into the prefrontal cortex, while a global reduction in 5HT impairs inhibition, suggesting an interaction between dopaminergic and serotonergic systems in response inhibition (Harrison et al., 1997; Granon et al., 2000; Winstanley et al., 2004; van Gaalen et al., 2006; Bari et al., 2011). Robbins (2000) noted that in marmosets manipulations of dopamine and noradrenaline tend to produce effects on tasks predominantly engaging the dorsolateral prefrontal cortex, but manipulations of the serotonergic system tend to alter performance in tests sensitive to orbitofrontal dysfunction. Interestingly, a recent study from our group (Choudhury et al., 2019) showed a significant reduction in SSRT after treatment with the dopamine precursor levodopa in Parkinson’s patients, potentially supporting the contention that movement stopping is mediated by circuits involving the dorsolateral prefrontal cortex.

Previous work (Deakin et al., 2004; Acheson et al., 2006) found that behavioral inhibition in a decision-making task was only impaired at higher doses of diazepam (20 mg) and not at typical therapeutic doses of 5–10 mg. However, in our study even therapeutic doses of diazepam could impair response inhibition in healthy individuals, without compromising RT. This was presumably by modulating GABA_A_ receptors in the frontal cortex or the basal ganglia.

Rather than manipulating GABA_A_ efficacy as in this study, Hermans et al. (2018) measured endogenous GABA levels in the brain using magnetic resonance spectroscopy. Older adults had lower levels of GABA, and also slower SSRT than younger participants. The association between lower GABA and slower SSRT was also seen just within the older subject group. Chowdhury et al. (2019) used transcranial magnetic brain stimulation to measure short-interval intracortical inhibition (SICI) and also concluded that lower inhibition was associated with slower SSRT. The direction of this association is opposite to that which we observed: enhancing GABA_A_ efficacy in our study led to slower SSRT. However, it should be remembered that GABAergic networks are far from simple. For example, in the cerebral cortex, GABAergic cells expressing vasoactive intestinal polypeptide (VIP) inhibit those expressing somatostatin, which in turn inhibit excitatory pyramidal neurons (Karnani et al., 2016). Both inhibition and disinhibition will be potentiated by diazepam. The level of GABA measured by magnetic resonance spectroscopy is a composite of the contributions from all inhibitory circuits in a given region; SICI measures inhibition of corticospinal pyramidal neurons. Differences in the sensitivity of circuits to each approach likely underlie the different direction of effects seen.

There are some reports of non-GABA_A_ receptor occupancy (5HT, D2) by diazepam (Saner and Pletscher, 1979; Gomez et al., 2017; van der Kooij et al., 2018) raising the possibility that the effects we observed could be mediated *via* non-GABAergic mechanisms. However, this is unlikely to explain our results given that we observed effects at therapeutic doses of diazepam and that GABAergic networks are a crucial substrate of response inhibition (Nicholson et al., 2018).

## Conclusion

Our results suggest that therapeutic doses of diazepam can significantly alter response inhibition. Inappropriate responses in a stop signal task are presumably mediated by inhibition of the prefrontal cortex, through GABAergic mechanisms. These changes occurred at doses that had no effect on the simple RT. This indicates that even a therapeutic dose of diazepam should be taken with adequate precaution, especially in cases where motor compromise is already a feature.

## Data Availability Statement

The raw data supporting the conclusions of this article will be made available by the authors on request, without undue reservation.

## Ethics Statement

The studies involving human participants were reviewed and approved by Institutional Ethics Committee (reference number I-NK/IEC/99/2019 ver.1. dated 29 April 2019) Institute of Neurosciences Kolkata, Kolkata, India. The patients/participants provided their written informed consent to participate in this study.

## Author Contributions

SS and SC: study concept and design, acquisition of data, analysis, and interpretation, writing the first draft, and critical revision of the manuscript for important intellectual content. NI: acquisition of data, analysis, and interpretation, and critical revision of the manuscript for important intellectual content. MC and MdC: critical revision of the manuscript for important intellectual content. MB: study concept and design, acquisition of data, analysis, and interpretation, and critical revision of the manuscript for important intellectual content. SB: study concept and design, analysis and interpretation, critical revision of the manuscript for important intellectual content, and study supervision. HK: study concept and design, acquisition of data, analysis, and interpretation, critical revision of the manuscript for important intellectual content, and study supervision. All authors contributed to the article and approved the submitted version.

## Conflict of Interest

The authors declare that the research was conducted in the absence of any commercial or financial relationships that could be construed as a potential conflict of interest.

## References

[B1] Acheson A. Reynolds B. Richards J. B. de Wit H. (2006). Diazepam impairs behavioral inhibition but not delay discounting or risk taking in healthy adults. Exp. Clin. Psychopharmacol. 14, 190–198. 10.1037/1064-1297.14.2.190 16756423

[B3] Aron A. R. Behrens T. E. Smith S. Frank M. J. Poldrack R. A. (2007). Triangulating a cognitive control network using diffusion-weighted magnetic resonance imaging (MRI) and functional MRI. J. Neurosci. 27, 3743–3752. 10.1523/JNEUROSCI.0519-07.2007 17409238 PMC6672420

[B2] Aron A. R. Poldrack R. A. (2006). Cortical and subcortical contributions to Stop signal response inhibition: role of the subthalamic nucleus. J. Neurosci. 26, 2424–2433. 10.1523/JNEUROSCI.4682-05.2006 16510720 PMC6793670

[B4] Aron A. R. Robbins T. W. Poldrack R. A. (2014). Inhibition and the right inferior frontal cortex: one decade on. Trends Cogn. Sci. 18, 177–185. 10.1016/j.tics.2013.12.003 24440116

[B5] Ascoli G. A. Alonso-Nanclares L. Anderson S. A. Barrionuevo G. Benavides-Piccione R. Burkhalter A.. (2008). Petilla terminology: nomenclature of features of GABAergic interneurons of the cerebral cortex. Nat. Rev. Neurosci. 9, 557–568. 10.1038/nrn2402 18568015 PMC2868386

[B6] Baker S. C. Rogers R. D. Owen A. M. Frith C. D. Dolan R. J. Frackowiak R. S. J. (1996). Neural systems engaged by planning: a PET study of the Tower of London task. Neuropsychologia 34, 515–526. 10.1016/0028-3932(95)00133-6 8736565

[B7] Bari A. Mar A. C. Theobald D. E. Elands S. A. Oganya K. C. Eagle D. M.. (2011). Prefrontal and monoaminergic contributions to stop-signal task performance in rats. J. Neurosci. 31, 9254–9263. 10.1523/JNEUROSCI.1543-11.2011 21697375 PMC3145112

[B8] Bechara A. Noel X. Crone E. A. (2006). “Loss of willpower: abnormal neural mechanisms of impulse control and decision making in addiction,” in Handbook of Implicit Cognition and Addiction, eds Wiers R. W. Stacy A. W. (Thousand Oaks, CA: Sage Publications, Inc.), 215–232.

[B9] Boehler C. N. Schevernels H. Hopf J. M. Stoppel C. M. Krebs R. M. (2014). Reward prospect rapidly speeds up response inhibition *via* reactive control. Cogn. Affect. Behav. Neurosci. 14, 593–609. 10.3758/s13415-014-0251-5 24448735

[B10] Choudhury S. Roy A. Mondal B. Singh R. Halder S. Chatterjee K.. (2019). Slowed movement stopping in Parkinson’s disease and focal dystonia is improved by standard treatment. Sci. Rep. 9:19504. 10.1038/s41598-019-55321-5 31862983 PMC6925208

[B11] Chowdhury N. S. Livesey E. J. Harris J. A. (2019). Individual differences in intracortical inhibition during behavioural inhibition. Neuropsychologia 124, 55–65. 10.1016/j.neuropsychologia.2019.01.008 30654018

[B12] Dalley J. W. Fryer T. D. Brichard L. Robinson E. S. Theobald D. E. Laane K.. (2007). Nucleus accumbens D2/3 receptors predict trait impulsivity and cocaine reinforcement. Science 315, 1267–1270. 10.1126/science.1137073 17332411 PMC1892797

[B14] Deakin J. B. Aitken M. R. Dowson J. H. Robbins T. W. Sahakian B. J. (2004). Diazepam produces disinhibitory cognitive effects in male volunteers. Psychopharmacology 173, 88–97. 10.1007/s00213-003-1695-4 14689162

[B15] Debey E. De Schryver M. Logan G. D. Suchotzki K. Verschuere B. (2015). From junior to senior Pinocchio: a cross-sectional lifespan investigation of deception. Acta Psychol. 160, 58–68. 10.1016/j.actpsy.2015.06.007 26182909

[B13] De Wit H. Richards J. B. (2004). Dual determinants of drug use in humans: rewards and impulsivity. Nebr. Sym. Motiv. 50, 19–55. 15160637

[B16] Downing S. S. Lee Y. T. Farb D. H. Gibbs T. T. (2005). Benzodiazepine modulation of partial agonist efficacy and spontaneously active GABA^(A)^ receptors supports an allosteric model of modulation. Br. J. Pharmacol. 145, 894–906. 10.1038/sj.bjp.0706251 15912137 PMC1576208

[B17] Eagle D. M. Tufft M. R. Goodchild H. L. Robbins T. W. (2007). Differential effects of modafinil and methylphenidate on stop-signal reaction time task performance in the rat and interactions with the dopamine receptor antagonist cis-flupenthixol. Psychopharmacology 192, 193–206. 10.1007/s00213-007-0701-7 17277934

[B18] Ersche K. D. Jones P. S. Williams G. B. Turton A. J. Robbins T. W. Bullmore E. T. (2012). Abnormal brain structure implicated in stimulant drug addiction. Science 335, 601–604. 10.1126/science.1214463 22301321

[B19] Fillmore M. T. Rush C. R. L H. (2002). Acute effects of oral cocaine on inhibitory control of behavior in humans. Drug Alcohol Depend. 67, 157–167. 10.1016/s0376-8716(02)00062-5 12095665

[B20] Ford R. D. Rech R. H. Commissaris R. L. Meyer L. Y. (1979). Effects of acute and chronic interactions of diazepam and d-amphetamine on punished behavior of rats. Psychopharmacology 65, 197–204. 10.1007/bf00433049 117489

[B21] Forstmann B. U. Keuken M. C. Jahfari S. Bazin P. L. Neumann J. Schäfer A.. (2012). Cortico-subthalamic white matter tract strength predicts interindividual efficacy in stopping a motor response. NeuroImage 60, 370–375. 10.1016/j.neuroimage.2011.12.044 22227131

[B22] Frank M. J. (2006). Hold your horses: a dynamic computational role for the subthalamic nucleus in decision making. Neural Netw. 19, 1120–1136. 10.1016/j.neunet.2006.03.006 16945502

[B23] Gelkopf M. Bleich A. Hayward R. Bodner G. Adelson M. (1999). Characteristics of benzodiazepine abuse in methadone maintenance treatment patients: a 1 year prospective study in an Israeli clinic. Drug Alcohol Depend. 55, 63–68. 10.1016/s0376-8716(98)00175-6 10402150

[B24] Gielen M. C. Lumb M. J. Smart T. G. (2012). Benzodiazepines modulate GABA_A_ receptors by regulating the preactivation step after GABA binding. J. Neurosci. 32, 5707–5715. 10.1523/JNEUROSCI.5663-11.2012 22539833 PMC6703631

[B25] Gomez A. A. Fiorenza A. M. Boschen S. L. Sugi A. H. Beckman D. Ferreira S. T.. (2017). Diazepam inhibits electrically evoked and tonic dopamine release in the nucleus accumbens and reverses the effect of amphetamine. ACS Chem. Neurosci. 8, 300–309. 10.1021/acschemneuro.6b00358 28038309

[B26] Granon S. Passetti F. Thomas K. L. Dalley J. W. Everitt B. J. Robbins T. W. (2000). Enhanced and impaired attentional performance after infusion of D1 dopaminergic receptor agents into rat prefrontal cortex. J. Neurosci. 20, 1208–1215. 10.1523/JNEUROSCI.20-03-01208.2000 10648725 PMC6774157

[B52] Hanes D. P. Schall J. D. (1996). Neural control of voluntary movement initiation. Science 274, 427–430. 10.1126/science.274.5286.427 8832893

[B27] Harrison A. A. Everitt B. J. Robbins T. W. (1997). Central 5-HT depletion enhances impulsive responding without affecting the accuracy of attentional performance: interactions with dopaminergic mechanisms. Psychopharmacology 133, 329–342. 10.1007/s002130050410 9372531

[B28] Heishman S. J. Arasteh K. Stitzer M. L. (1997). Comparative effects of alcohol and marijuana on mood, memory, and performance. Pharmacol. Biochem. Behav. 58, 93–101. 10.1016/s0091-3057(96)00456-x 9264076

[B29] Hermans L. Leunissen I. Pauwels L. Cuypers K. Peeters R. Puts N. A. J.. (2018). Brain GABA levels are associated with inhibitory control deficits in older adults. J. Neurosci. 38, 7844–7851. 10.1523/JNEUROSCI.0760-18.2018 30064995 PMC6125814

[B30] Hikosaka O. Isoda M. (2010). Switching from automatic to controlled behavior: cortico-basal ganglia mechanisms. Trends Cogn. Sci. 14, 154–161. 10.1016/j.tics.2010.01.006 20181509 PMC2847883

[B31] Inase M. Tokuno H. Nambu A. Akazawa T. Takada M. (1999). Corticostriatal and corticosubthalamic input zones from the presupplementary motor area in the macaque monkey: comparison with the input zones from the supplementary motor area. Brain Res. 833, 191–201. 10.1016/s0006-8993(99)01531-0 10375694

[B32] Karnani M. M. Jackson J. Ayzenshtat I. Hamzehei Sichani A. Manoocheri K. Kim S.. (2016). Opening holes in the blanket of inhibition: localized lateral disinhibition by VIP interneurons. J. Neurosci. 36, 3471–3480. 10.1523/JNEUROSCI.3646-15.2016 27013676 PMC4804006

[B34] Leunissen I. Zandbelt B. B. Potocanac Z. Swinnen S. P. Coxon J. P. (2017). Reliable estimation of inhibitory efficiency: to anticipate, choose or simply react? Eur. J. Neurosci. 45, 1512–1523. 10.1111/ejn.13590 28449195

[B35] Lévesque J. C. Parent A. (2005). GABAergic interneurons in human subthalamic nucleus. Mov. Disord. 20, 574–584. 10.1002/mds.20374 15645534

[B36] Li C. S. Yan P. Sinha R. Lee T. W. (2008). Subcortical processes of motor response inhibition during a stop signal task. NeuroImage 41, 1352–1363. 10.1016/j.neuroimage.2008.04.023 18485743 PMC2474693

[B37] Ljungberg T. (1990). Diazepam and decision making in the rat: negative evidence for reduced tolerance to reward delay. Psychopharmacology 102, 117–121. 10.1007/bf02245755 2392499

[B38] Ljungberg T. Lidfors L. Enquist M. Ungerstedt U. (1987). Impairment of decision making in rats by diazepam: implications for the “anticonflict” effects of benzodiazepines. Psychopharmacology 92, 416–423. 10.1007/bf00176471 2888151

[B39] Logan G. D. Cowan W. B. Davis K. A. (1984). On the ability to inhibit simple and choice reaction time responses: a model and a method. J. Exp. Psychol. 10, 276–291. 10.1037/0096-1523.10.2.276 6232345

[B40] Mandelli M. Tognoni G. Garattini S. (1978). Clinical pharmacokinetics of diazepam. Clin. Pharmacokinet. 3, 72–91. 10.2165/00003088-197803010-00005 346285

[B41] Manes F. Sahakian B. Clark L. Rogers R. Antoun N. Aitken M. R.. (2002). Decision-making processes following damage to the prefrontal cortex. Brain 125, 624–639. 10.1093/brain/awf049 11872618

[B42] Möhler H. (2015). The legacy of the benzodiazepine receptor: from flumazenil to enhancing cognition in Down syndrome and social interaction in autism. Adv. Pharmacol. 72, 1–36. 10.1016/bs.apha.2014.10.008 25600365

[B43] Mückschel M. Stock A. K. Beste C. (2014). Psychophysiological mechanisms of interindividual differences in goal activation modes during action cascading. Cereb. Cortex 24, 2120–2129. 10.1093/cercor/bht066 23492952

[B44] Munakata Y. Herd S. A. Chatham C. H. Depue B. E. Banich M. T. O’Reilly R. C. (2011). A unified framework for inhibitory control. Trends Cogn. Sci. 15, 453–459. 10.1016/j.tics.2011.07.011 21889391 PMC3189388

[B45] Nicholson M. W. Sweeney A. Pekle E. Alam S. Ali A. B. Duchen M.. (2018). Diazepam-induced loss of inhibitory synapses mediated by PLCdelta/ Ca^2+^/calcineurin signalling downstream of GABA_A_ receptors. Mol. Psychiatry 23, 1851–1867. 10.1038/s41380-018-0100-y 29904150 PMC6232101

[B46] Reynolds B. Richards J. B. Dassinger M. de Wit H. (2004). Therapeutic doses of diazepam do not alter impulsive behavior in humans. Pharmacol. Biochem. Behav. 79, 17–24. 10.1016/j.pbb.2004.06.011 15388279

[B47] Robbins T. W. (2000). Chemical neuromodulation of frontal-executive functions in humans and other animals. Exp. Brain Res. 133, 130–138. 10.1007/s002210000407 10933217

[B48] Rogers R. D. Owen A. M. Middleton H. C. Williams E. J. Pickard J. D. Sahakian B. J.. (1999). Choosing between small, likely rewards and large, unlikely rewards activates inferior and orbital prefrontal cortex. J. Neurosci. 20, 9029–9038. 10.1523/JNEUROSCI.19-20-09029.1999 10516320 PMC6782753

[B49] Rudolph U. Knoflach F. (2011). Beyond classical benzodiazepines: novel therapeutic potential of GABA_A_ receptor subtypes. Nat. Rev. Drug Discov. 10, 685–697. 10.1038/nrd3502 21799515 PMC3375401

[B50] Saner A. Pletscher A. (1979). Effect Of diazepam on cerebral 5-hydroxytryptamine synthesis. Eur. J. Pharmacol. 55, 315–318. 10.1016/0014-2999(79)90200-0 313340

[B51] Schachar R. Logan G. D. (1990). Impulsivity and inhibitory control in normal development and childhood psychopathology. Dev. Psychol. 26, 710–720. 10.1037/0012-1649.26.5.710

[B53] Tannock R. Schachar R. Logan G. D. (1995). Methylphenidate and cognitive flexibility: dissociated dose effects in hyperactive children. J. Abnorm. Child Psychol. 23, 235–266. 10.1007/bf01447091 7642836

[B33] van der Kooij M. A. Hollis F. Lozano L. Zalachoras I. Abad S. Zanoletti O.. (2018). Diazepam actions in the VTA enhance social dominance and mitochondrial function in the nucleus accumbens by activation of dopamine D1 receptors. Mol. Psychiatry 23, 569–578. 10.1038/mp.2017.135 28727688 PMC5822450

[B54] van Gaalen M. M. Brueggeman R. J. Bronius P. F. Schoffelmeer A. N. Vanderschuren L. J. (2006). Behavioral disinhibition requires dopamine receptor activation. Psychopharmacology 187, 73–85. 10.1007/s00213-006-0396-1 16767417

[B55] Verbruggen F. Chambers C. D. Logan G. D. (2013). Fictitious inhibitory differences: how skewness and slowing distort the estimation of stopping latencies. Psychol. Sci. 24, 352–362. 10.1177/0956797612457390 23399493 PMC3724271

[B56] Verbruggen F. Stevens T. Chambers C. D. (2014). Proactive and reactive stopping when distracted: an attentional account. J. Exp. Psychol. Hum. Percept. Perform. 40, 1295–1300. 10.1037/a0036542 24842070 PMC4120704

[B58] Whelan R. Conrod P. J. Poline J. B. Lourdusamy A. Banaschewski T. Barker G. J.. (2012). Adolescent impulsivity phenotypes characterized by distinct brain networks. Nat. Neurosci. 15, 920–925. 10.1038/nn.3092 22544311

[B59] Winstanley C. A. Theobald D. E. Dalley J. W. Glennon J. C. Robbins T. W. (2004). 5-HT2A and 5-HT2C receptor antagonists have opposing effects on a measure of impulsivity: interactions with global 5-HT depletion. Psychopharmacology 176, 376–385. 10.1007/s00213-004-1884-9 15232674

[B60] Woods J. R. Plessinger M. A. Clark K. E. (1987). Effect of cocaine on uterine blood flow and fetal oxygenation. JAMA 257, 957–961. 10.1001/jama.1987.03390070077027 3806879

